# Successful management of portal vein thrombosis in a Yorkshire Terrier with protein-losing enteropathy

**DOI:** 10.1186/s12917-020-02632-9

**Published:** 2020-11-02

**Authors:** Yumi Sakamoto, Kumiko Ishigaki, Chieko Ishikawa, Tomohiro Nakayama, Kazushi Asano, Manabu Sakai

**Affiliations:** 1 Department of Veterinary Medicine, College of Bioresource Sciences, Nihon University, 1866 Kameino, Kanagawa 252-0880 Fujisawa, Japan; 2 Laboratory of Veterinary Hepatology & Gastroenterology, Depertment of Veterinary Medicine, College of Bioresource Sciences, Nihon University, 1866 Kameino, Kanagawa 252-0880 Fujisawa, Japan

**Keywords:** canine, computed tomography, portal hypertension, rivaroxaban

## Abstract

**Background:**

Portal vein thrombosis (PVT) is a rare presentation in dogs with protein-losing enteropathy (PLE). Rivaroxaban, an oral, selective, direct factor Xa inhibitor, has not been reported to be administrated for canine PVT and the effect is unclear in dogs with PLE.

**Case presentation:**

An 11-year-old Yorkshire Terrier presented with moderate ascites. The dog had severe hypoalbuminemia (1.2 g/dL), and a portal vein thrombus was confirmed on computed tomographic angiography (CTA). On endoscopic examination, it became apparent that the hypoalbuminemia was caused by PLE, which was consequent of lymphatic dilation and lymphoplasmacytic enteritis. Therefore, the dog was initially treated with oral administrations of spironolactone and clopidogrel, with dietary fat restriction. However, a follow-up CTA showed no changes in the ascites, thrombus, and portal vein to aorta (PV/Ao) ratio. Therefore, the dog was additionally prescribed rivaroxaban and low-dose prednisolone for the portal vein thrombus and hypoalbuminemia due to lymphoplasmacytic enteritis, respectively. Following the treatment, the PV/Ao ratio decreased because of a decrease in the thrombus and the ascites disappeared completely with an elevation of albumin concentration (1.9 g/dL).

**Conclusions:**

This case report demonstrated that oral administration of rivaroxaban combined with low-dose glucocorticoid was effective management for PVT in a dog with PLE.

## Background

Portal vein thrombosis (PVT) due to thrombosis within the extrahepatic portal venous system is an uncommon occurrence. It mainly develops in dogs with hypercoagulability due to glucocorticoid therapy, hepatic diseases, including chronic hepatitis, congenital portosystemic shunt (CPSS), and in dogs with protein-losing enteropathy (PLE) [1–4]. In addition, chronic PVT may present with ascites and acquired portosystemic collaterals (APSCs) due to prehepatic portal hypertension (PH) [5]. Recently, in dogs with pancreatitis, computed tomographic angiography (CTA) was used to diagnose PVT [6, 7]. Furthermore, CTA can clearly identify the presence of APSCs and help to estimate the portal vein to aorta (PV/Ao) ratio [8]. Therefore, CTA is a powerful and accurate tool for identifying canine PVT and PH.

The management of PVT in dogs has been attempted with anticoagulants and antiplatelet or thrombolytic therapy, including low-dose aspirin, low molecular weight heparin, unfractionated heparin, warfarin, and clopidogrel [1]. Recently, rivaroxaban, a direct oral anticoagulant, was used in dogs with thromboembolic disease and was confirmed to be efficacious for thrombotic complications [9–12]. However, rivaroxaban therapy has not been reported in dogs with PVT. This case describes the successful management of PVT due to PLE in a Yorkshire Terrier with the administration of rivaroxaban and low-dose glucocorticoid.

## Case presentation

An 11-year-old female spayed Yorkshire Terrier, weighing 3.2 kg, was presented to our hospital with a one-month history of abdominal swelling. Seven years ago, the dog was diagnosed with a single extrahepatic CPSS based on CTA findings and underwent surgical attenuation of the shunt with an ameroid constrictor in our hospital. A follow-up evaluation was performed by the referring veterinarian who reported no recurrence of clinical signs and complications.

**Fig. 1:**
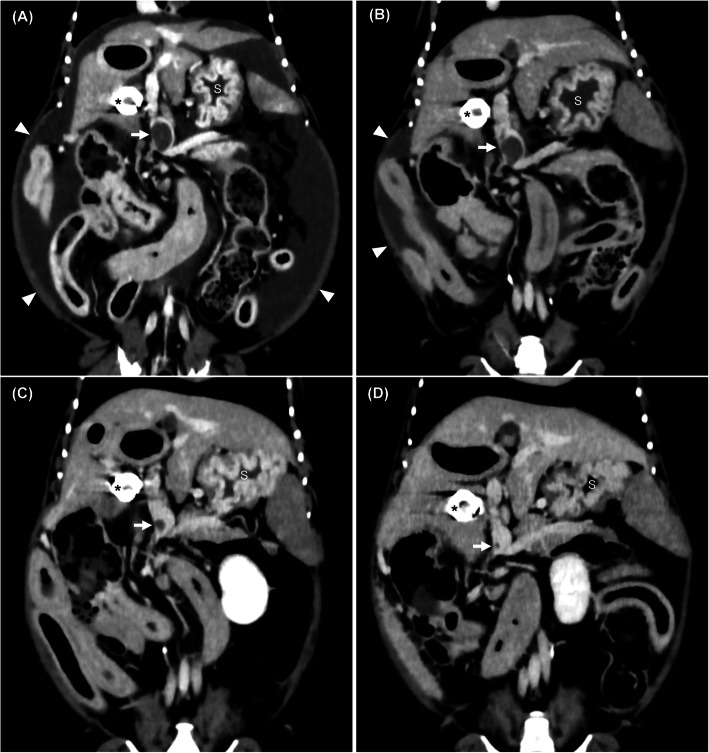
CTA images of a Yorkshire Terrier on day 1 (**a**), day 35 (**b**), day 182 (**c**), and day 373 (**d**). The size of the thrombus in the main portal vein (arrow) remained unchanged; the volume of ascites (arrowheads) before (**a**) and after (**b**) treatment with clopidogrel and spironolactone. On the two CTA images acquired after combined therapy with rivaroxaban and prednisolone (**c**, **d**), ascites was not detected and the thrombus size appears to have gradually reduced in the main portal vein (arrow). Asterisk, ameroid constrictor; S, stomach; CTA, computed tomographic angiography

At presentation, the dog was fine and bright without digestive signs. On physical examination, the abdomen was distended and vital signs were normal, including a body temperature of 38.4 °C, a heart rate of 120 bpm, and a respiratory rate of 24 breaths per minute. Hematological findings included white blood cells count (WBC) of 10,300/µL (reference range, 6,000–17,000/µL), red blood cells count (RBC) of 8.11 M/µL (reference range, 5.50–8.50 M/µL), slightly elevated packed cell volume (PCV) of 58% (reference range, 37–55%), and platelets of 221,000/µL (reference range, 200,000–500,000/µL). Results of the routine biochemical analysis revealed mildly elevated levels of aspartate transaminase (AST 60 U/L; reference range, 0–50 U/L), severe hypoproteinemia (3.1 g/dL; reference range, 5.2–8.2 g/dL) with severe hypoalbuminemia (1.2 g/dL; reference range, 2.7–3.8 g/dL), and mild hypocholesterolemia (109 mg/dL; reference range, 110–320 mg/dL). The alanine aminotransferase (ALT 47 U/L; reference range, 10–100 U/L), alkaline phosphatase (ALP 48 U/L; reference range, 23–212U/L), and γ-glutamyl transpeptidase (GGT 4 U/L; reference range, 0–7 U/L) levels were normal. Blood coagulation screening tests revealed normal prothrombin time (PT 5.1 s; reference range, 4.0–6.0 s), slight prolonged activated partial thromboplastin time (APTT 17.1 s; reference range, 10.0–16.0 s), and normal fibrinogen concentration (217.9 mg/dL; reference range, 86.0–375.0 mg/dL). Plasma D-Dimer concentration was high (4.5 µg/mL; reference range, < 2.0 µg/mL) and antithrombin (AT) activity was reduced (82%; reference range, 102–156%). Urine protein-to-creatinine ratio was normal (0.11; reference range, 0.00–0.50, urine protein 4.7 mg/dL, urine creatinine 42.9 mg/dL). No abnormalities were detected on thoracic and abdominal radiographic examinations. Abdominal ultrasound revealed moderate amounts of anechoic, free peritoneal fluid, and an 8.7 × 14.3-mm diameter hypoechoic intraluminal mass was present in the main portal vein. The liver parenchyma showed no abnormalities. Results of the cytological analysis of peritoneal fluid were consistent with a low protein transudate (total protein 0.2 g/dL, specific gravity 1.006, nucleated cell counts 200/µL). To confirm the presence of a mass in the main portal vein, CTA was performed with a 320-detector row computed tomography (CT) scanner (Aquilion One, Canon medical systems, Tochigi, Japan). The CTA findings identified dilation of the main portal vein, a filling defect in the center of the vessel, and ascites (Fig. 1a). No other abnormalities were detected in the systemic circulation and organs on thoracic and abdominal CTA images. Moreover, there was no evidence for the development of APSCs. In addition, the PV/Ao ratio was calculated to be 3.56 with curved planar reformation images acquired by a CT scan as previously reported [8]. Based on the CTA results, the dog was diagnosed with prehepatic PH due to a portal vein thrombus. The dog was treated with spironolactone (0.5 mg/kg PO q12) and clopidogrel (2 mg/kg PO q24).

**Fig. 2:**
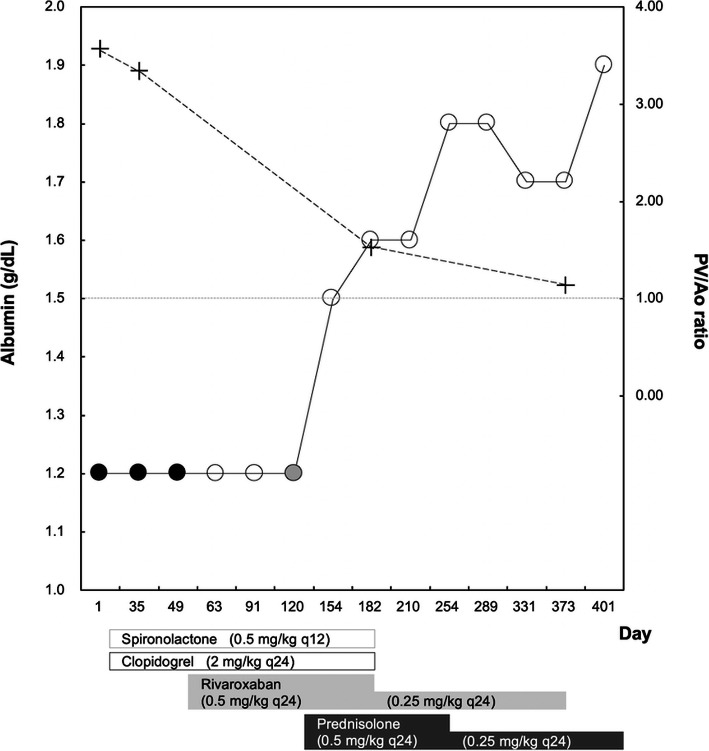
Time course of changes in serum albumin concentration (circles) and portal vein to aorta (PV/Ao) ratio (crosses) in a Yorkshire Terrier with protein-losing enteropathy and portal vein thrombosis treated with spironolactone, clopidogrel, rivaroxaban, and prednisolone. Black circles, moderate ascites; gray circle, mild ascites; open circles, no-ascites

**Fig. 3:**
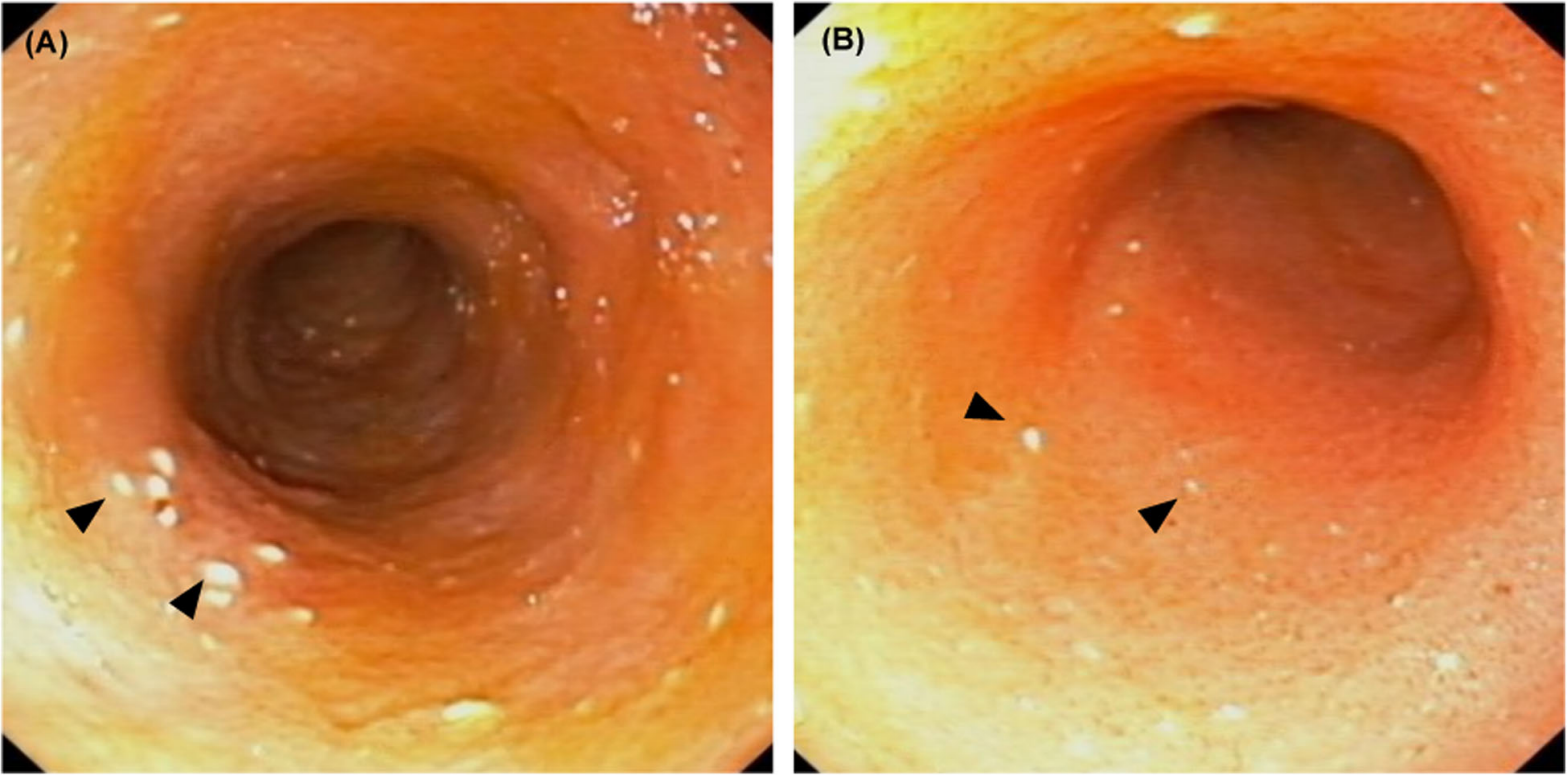
Endoscopic findings of the duodenum in a Yorkshire Terrier with protein-losing enteropathy on day 35 (**a**) and day 373 (**b**). The number of pinpoint white foci (arrowheads) in the mucosal edema appeared to be slightly reduced after treatment with prednisolone

On day 35, the dog had no clinical signs and physical examination revealed a body weight of 3.0 kg and moderate abdominal distention. No abnormalities were detected on hematology (WBC 10,700/µL; RBC 7.61 M/µL; PCV 52%; platelets 272,000/µL). Biochemical abnormalities were detected on follow-up, including hypoproteinemia (3.2 g/dL), hypoalbuminemia (1.2 g/dL, Fig. 2), and hypocholesterolemia (89 mg/dL). Other results were normal (ALT 49 U/L, AST 45 U/L, ALP 37 U/L, GGT 4 U/L). Blood coagulation screening tests were normal (PT 5.8 s, APTT 15.4 s, fibrinogen concentration 201.2 mg/dL). Plasma D-Dimer concentration was high (3.2 µg/mL) and AT activity was normal (106%). Therefore, the dog was suspected to have PLE and an endoscopy-guided intestinal biopsy was performed on day 35 for definitive diagnosis by histopathological examination. Simultaneously, PVT was reassessed by CTA. On endoscopic examination, mucosal edema and pinpoint white foci were observed in the duodenum (Fig. 3a). Moreover, the size of the main portal vein thrombus, the PV/Ao ratio was 3.34 (Fig. 2), and the volume of ascites on CTA findings almost unchanged (Fig. 1b). Histopathologically, the duodenal biopsy specimens were characterized by moderate dilation of the lymphatics and mild infiltration of chronic inflammatory cells in the submucosae, mainly lymphocytes and plasma cells. The final histopathological diagnosis was intestinal lymphangiectasia and lymphoplasmacytic enteritis.

On day 49, the dog presented with moderate ascites. The canine chronic enteropathy clinical activity index (CCECAI) for assessment of the clinical severity was 6, according to a previously established scoring system [13]. The patient was suspected to have a persistent hypercoagulable state because of decreased AT activity (87%) and elevated D-Dimer concentration (3.2 µg/mL). Therefore, glucocorticoid therapy for lymphoplasmacytic enteritis was avoided and rivaroxaban therapy (0.5 mg/kg PO q24) was initiated. In addition, a dietary fat restriction was advised for hypoalbuminemia (1.2 g/dL) and ascites due to PLE as previously reported [14]. On day 63 follow-up, the abdominal distension improved and ultrasonography findings did not show any ascites. However, clinicopathological abnormalities were still detected, including hypoalbuminemia (1.2 g/dL), decreased AT activity (94%), and elevated D-Dimer concentration (6.4 µg/mL). Moreover, abdominal ultrasound showed the persistent presence of the main portal vein thrombus. Therefore, rivaroxaban therapy was continued at the same dosage.

On day 120, the dog presented with the recurrence of mild ascites and severe hypoalbuminemia (1.2 g/dL). Additionally, AT activity (96%) and D-Dimer concentration (7.5 µg/mL) remained abnormal. Based on these findings, the dog was considered unresponsive to dietary fat restriction for intestinal lymphangiectasia. It was suspected that the degree of inflammation was progressing more than before. Therefore, prednisolone (0.5 mg/kg PO q24) was added to the existing regimen to treat lymphoplasmacytic enteritis. Following glucocorticoid therapy, on day 154, serum albumin concentration slightly increased to 1.5 g/dL and the abdominal distention improved without the presence of ascites. On day 182, the dog underwent a CTA for the reexamination of the portal vein thrombus. At this time, serum albumin concentration was maintained at 1.6 g/dL and D-Dimer concentration returned to the normal range (0.0 µg/mL). The CTA findings showed a complete resolution of the ascites, and the size of the main portal vein thrombus was smaller than before and PV/Ao ratio was 1.53 (Fig. 1c). Therefore, spironolactone was discontinued and the dosage of rivaroxaban was decreased (0.25 mg/kg PO q24). On day 254, serum albumin concentration had increased to 1.8 g/dL; thus, the dosage of prednisolone was decreased (0.25 mg/kg PO q24).

On day 373, the dog had a CTA and endoscopic reexamination for the PVT and PLE. The CCECAI score was 1 and the levels of albumin and D-Dimer were maintained at 1.7 g/dL and 0.1 µg/mL, respectively. CTA findings showed that the ascites completely disappeared, the thrombus size reduced, and PV/Ao ratio decreased (1.14, Fig. 1d). Endoscopic examination findings showed slightly pinpoint white foci in the duodenum (Fig. 3b). Results of histopathological examination of the duodenum biopsy samples confirmed a diagnosis of intestinal lymphangiectasia and lymphoplasmacytic enteritis. Based on these findings, the administration of rivaroxaban was discontinued. On day 401, the dog was in good general condition without ascites and the main portal vein thrombus under abdominal ultrasound. At the time of writing this article, the dog had no clinical abnormalities or side effects of the low-dose prednisolone therapy (0.25 mg/kg PO q24) and dietary fat restriction.

## Discussion and conclusions

Canine PLE is a common syndrome characterized by a loss of serum albumin across the intestinal wall due to inflammatory bowel disease, intestinal lymphoma, or intestinal lymphangiectasia [15]. Yorkshire Terrier is one of the breeds predisposed to non-neoplastic PLE [16, 17]. PLE has previously been reported to cause a thromboembolic event in dogs [1, 18, 19]. The enteric loss of AT concurrently with albumin mechanism is not the only cause of hypercoagulability of canine PLE [18]. Although the Yorkshire Terrier with PLE described herein had persistently low AT activity, the patient had borderline low AT activity due to PLE. Therefore, the involvement of other hypercoagulable factors, besides low AT activity, was suggested. Humans with inflammatory bowel disease have a high risk of systemic thromboembolism in the venous and arterial circulations [20, 21]. As our patient was also diagnosed with lymphoplasmacytic enteritis on histopathological examination, the inflammation might be associated with a hypercoagulable state. In a previous study of 33 dogs with PVT, two dogs had CPSS [1]. Our dog had a history of surgical attenuation of extrahepatic CPSS with an ameroid constrictor. Therefore, postoperative changes in blood flow in this dog might be associated with the thromboembolic event in the portal vein.

In a previous report of 33 cases with PVT, PLE and pancreatitis were only identified in one dog [1]. PVT was diagnosed in these dogs by ultrasound (27/33), necropsy (1/33), surgery (1/33), and CTA (1/33). Fortunately, in our case, a thrombus was identified within the main portal vein on the initial abdominal ultrasound examination. This may be due to the small breed of the dog and the thrombus being located at the level of the porta hepatis. However, it is difficult to visualize the portal vein in case of large breeds with deep-chested conformation, severe ascites, and interference by gas or food in the digestive tract [1]. Dogs with PLE frequently have abdominal distention due to severe ascites [15]. Recently, CTA, but not conventional ultrasound, has been used to diagnose PVT in dogs with pancreatitis [6, 7]. PVT might not be detected by ultrasound in dogs with PLE; therefore, we believe that CTA is a useful tool to detect PVT in dogs with PLE.

Rivaroxaban is an oral, selective, direct factor Xa inhibitor and is effective for the management of arterial or venous thrombotic disorders in humans [22]. According to the Consensus on the Rational Use of Antithrombotics in Veterinary Critical Case, [23] rivaroxaban at a recommended dose of 1–2 mg/kg/day is a safe and well-tolerated drug for use in dogs. Therefore, recently, it has been used as a new anticoagulant for the treatment of dogs with thrombotic complications such as pulmonary and systemic thromboembolism but without PVT [10–13]. In our patient, we first prescribed clopidogrel, an antiplatelet agent, for the treatment of PVT. However, the size of the portal vein thrombus remained unchanged on CTA images. Next, rivaroxaban therapy in combination with clopidogrel was initiated in our patient. Consequently, despite persistent hypoalbuminemia, the ascites disappeared on the follow-up abdominal ultrasound performed two weeks after the initiation of treatment. Thus, these findings suggest that rivaroxaban therapy may be effective for treating dogs with PVT and could decrease the resistance of blood flow into the portal vein. However, the dog had a recurrence of mild ascites due to severe hypoalbuminemia. Therefore, glucocorticoid therapy was initiated for further treatment of lymphoplasmacytic enteritis.

Conventionally, dogs with PLE are treated with steroids to induce immunosuppression [15]. However, glucocorticoid therapy is known to contribute to hypercoagulability and is associated with PVT in dogs [1, 2]. Thus, we initially avoided using prednisolone in our dog with PVT because of the risk of aggravation of the hypercoagulable state. Instead, we prescribed dietary fat restriction alone as the treatment for PLE. Dietary management is a potential strategy to treat PLE in Yorkshire Terriers [16]. Serum albumin concentrations in dogs with intestinal lymphangiectasia were increased by dietary fat restriction at 1 to 2 months after treatment, compared with that before treatment. [14] However, our patient’s serum albumin concentrations remained unchanged (1.2 g/dL) until day 120. Low dose prednisolone therapy was started because the PLE in our patient was suspected to progress to lymphoplasmacytic enteritis. After glucocorticoid treatment, serum albumin concentrations continued to increase to more than 1.5 g/dL and the dog had no recurrence of ascites ever since. Severe hypoalbuminemia (below 1.5 g/dL) usually leads to ascites in dogs without PH [24]. The presence of PVT is one of the main causes of canine prehepatic PH [5]. In our dog, however, there was no evidence for the development of APSCs concomitant with PH.

The clinical consequences of PH include ascites and the development of APSCs [5]. Although the dog had moderate ascites, there was no evidence of APSCs on CTA. The PV/Ao ratio is a noninvasive marker of increased portal vein pressure in dogs with PH [5]. According to a previous report that calculated PV/Ao ratio on helical CT images, the median PV/Ao ratio was 0.95 (0.80–1.15) in healthy dogs [8]. In our dog, the PV/Ao ratio remarkably increased to 3.56 and 3.34 on day 1 and day 35, respectively. In addition, despite severe hypoalbuminemia, the ascites improved transiently after the treatment of PVT with rivaroxaban. Therefore, although there was no evidence for the development of APSCs, the dog was strongly suspected to have prehepatic PH due to PVT. Thus, the ascites in this dog may have developed because of both severe hypoalbuminemia due to PLE and prehepatic PH due to PVT.

In conclusion, CTA was a useful tool for the diagnosis and monitoring of a thrombus in the portal vein and oral administration of rivaroxaban combined with low-dose glucocorticoid was effective management for PVT in a dog with PLE.

## Data Availability

All data generated or analyzed during this study are included in this published article.

## Abbreviations

ALP: alkaline phosphatase
ALT: alanine aminotransferase
APSCs: acquired portosystemic collaterals
APTT: activated partial thromboplastin time
AST: aspartate transaminase
AT: antithrombin
CCECAI: canine chronic enteropathy clinical activity index
CPSS: congenital portosystemic shunt
CT: computed tomography
CTA: computed tomographic angiography
GGT: γ-glutamyl transpeptidase
PCV: packed cell volume
PH: portal hypertension
PLE: protein-losing enteropathy
PT: prothrombin time
PV/Ao: portal vein to aorta
PVT: portal vein thrombosis
RBC: red blood cells count
WBC: white blood cells count
